# Mitochondrial Quality Control in Sarcopenia: Updated Overview of Mechanisms and Interventions

**DOI:** 10.14336/AD.2021.0427

**Published:** 2021-12-01

**Authors:** Di Liu, Yi-bin Fan, Xiao-hua Tao, Wei-li Pan, Yu-xiang Wu, Xiu-hua Wang, Yu-qiong He, Wen-feng Xiao, Yu-sheng Li

**Affiliations:** ^1^Department of Orthopaedics, Xiangya Hospital, Central South University, Changsha 410008, Hunan, China.; ^2^Department of Dermatology, Zhejiang provincial people’s hospital, People’s Hospital of Hangzhou Medical College, Hangzhou 310014, China.; ^3^School of Kinesiology, Jianghan University, Wuhan 430056, China.; ^4^Xiang Ya Nursing School, The Central South University, Changsha 410013, China.; ^5^National Clinical Research Center for Geriatric Disorders, Xiangya Hospital, Central South University, Changsha 410008, Hunan, China

**Keywords:** sarcopenia, mitochondria, mitochondrial quality control, therapeutic intervention

## Abstract

Sarcopenia is a common geriatric disorder characterized by decreased muscle strength, low muscle mass and poor physical performance. This aging-related skeletal muscle deterioration leads to adverse outcomes and severely impairs the quality of life of patients. The accumulation of dysfunctional mitochondria with aging is an important factor in the occurrence and progression of sarcopenia. Mitochondrial quality control (MQC) fundamentally ensures the normal mitochondrial functions and is comprised of four main parts: proteostasis, biogenesis, dynamics and autophagy. Therefore, any pathophysiologic factors compromising the quality control of homeostasis in the skeletal muscle may lead to sarcopenia. However, the specific theoretical aspects of these processes have not been fully elucidated. Current therapeutic interventions using nutritional and pharmaceutical treatments show a modest therapeutic efficacy; however, only physical exercise is recommended as the first-line therapy for sarcopenia, which can ameliorate skeletal muscle deficiency by maintaining the homeostatic MQC. In this review, we summarized the known mechanisms that contribute to the pathogenesis of sarcopenia by impairing normal mitochondrial functions and described potential interventions that mitigate sarcopenia through improving MQC.

## 1. Introduction

Senescence is a natural process of aging associated with degeneration of physical functions. The gradual loss of muscle mass, strength and function is one of the most important hallmarks of aging. Muscle mass starts to slightly decline from the age between 30 to 40 years of age along with a reduction in muscle function [1]. The reduction of muscle mass is more serious in populations with an inactive or sedentary lifestyle. The loss of muscle mass probably reaches 1 to 2% per year from 50 to 60 years and 3% to 5% per year at older ages [1]. In these people, 30% to 50% of the muscle mass may lose from 40 to 80 years totally [1]. Some of the cases may reach the diagnostic criteria of sarcopenia.

The term sarcopenia was coined by Irwin Rosenberg in the 1980s to describe an age-dependent decline in muscle mass and its adverse effects on human health [2]. In 2019, the European Working Group on Sarcopenia in Older People 2 (EWGSOP2) launched the latest diagnostic criteria for sarcopenia, including low muscle strength, decreased muscle quantity/quality or poor physical performance [3], highlighting the fundamental role of low muscle strength in the pathogenesis of sarcopenia. As a multifaceted geriatric disease characterized by progressive and generalized loss of skeletal muscle mass and function [4-6], the occurrence and progression of sarcopenia is always concomitant with various negative outcomes, including falls [7], fractures [8], loss of locomotion [9] and even mortality [10]. At the cellular and molecular levels, a constellation of latent mechanisms have been shown to participate in the sarcopenia, such as mitochondrial dysfunction[11], insulin resistance [12], inflammation [13], oxidative stress [14], adipose tissue infiltration [15] and neuromuscular impairment [16]. Notably, an increasing number of studies have indicated that dysfunctional mitochondria may play a central role in the pathogenesis of sarcopenia.

Mitochondria have strong impacts on the maintenance of cellular viability, including ATP production, oxidative phosphorylation (OXPHOX) homeostasis, calcium buffering and apoptosis. Therefore, healthy quality control is crucial for the preservation of intracellular homeostasis of muscle cells with aging. The MQC includes mitochondrial proteostasis, biogenesis, dynamics and autophagy [17, 18]. Orchestrated mechanisms contain several cellular factors and signaling pathways to ensure the integrity of mitochondria. Mitochondrial biogenesis is responsible for the generation of new mitochondria through the synergistic interaction of the nuclear and mitochondrial genes [19]; mitochondrial dynamics is achieved by continual transformation between fusion and fission to eliminate the accumulation of unhealthy mitochondria [20]; mitochondrial autophagy (mitophagy) is a process of selective removal of the hypofunctional and damaged mitochondria [21]. Adverse alternations in the quality control mechanisms may lead to mitochondrial dysfunction, which can further contribute to muscle wasting and even sarcopenia [22-25].

The incidence rate of sarcopenia in the mid-life and elderly population varies according to different age, operational definitions, regions and ethnicities [26-31]. A number of epidemiological studies have shown that the prevalence of sarcopenia gradually increases with age. It is conservatively estimated that 5%-13% of elderly individuals aged 60-70 years are suffering from sarcopenia. The numbers increase to 11%-50% among those aged 80 or above [32]. Since the number and proportion of the global aging population is rapidly growing, the socio-economic burden of individuals and society may increase due to higher prevalence of sarcopenia. Sarcopenia has been formally recognized as a disease with an ICD-10-CM (M62.84) code in 2016 [33, 34], which attracted additional attention for this degenerative disease. Physical activity is recommended as the primary treatment for sarcopenia to improve muscle strength and mass [35], although no specific drugs have been developed with therapeutic effects in sarcopenia. In this review, we summarized the potential mechanisms of mitochondrial dysfunction with an emphasis on promising therapeutic interventions to prevent and ameliorate sarcopenia during aging.

## 2. Mitochondrial Quality Control in Sarcopenia

**Figure 1.**
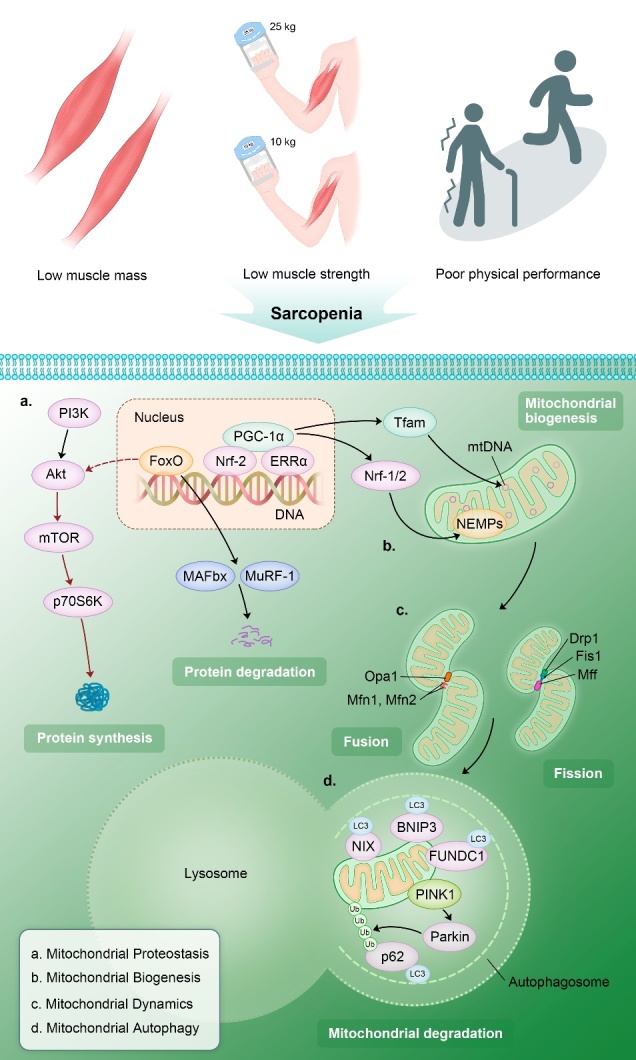
**The potential mechanisms of mitochondrial quality control (MQC) dyshomeostasis in sarcopenia.** Impaired mitochondrial proteostasis, biogenesis, dynamics and autophagy have been regarded as the major molecular mechanisms in mitochondrial dysfunction, which could lead to the onset and progression of sarcopenia. (PIK3: phosphoinositide 3-kinase; Akt: protein kinase B; mTOR: mechanistic target of rapamycin; FoxO: Forkhead Box O; MAFbx: muscle atrophy F-box; MuRF-1: muscle RING finger protein 1; PGC-1α: peroxisome proliferative activated receptor-γ coactivator-1α; Nrf-1 and 2: nuclear respiratory factor-1 and -2; ERRα: estrogen-related receptor alpha; Tfam: mitochondrial transcription factor A; NEMPs: nuclear-encoded mitochondrial proteins; mtDNA: mitochondrial DNA; Opa1: optic atrophy 1; Mfn1 and 2: mitofusin 1 and 2; Fis1: ﬁssion protein 1; Drp1: dynamin-related protein 1; Mff: mitochondrial ﬁssion factor; NIX: BCL2 interacting protein 3 like; BNIP3: BCL2 interacting protein 3; FUNDC1: FUN14 domain containing 1; PINK1: PTEN induced putative kinase 1; p62: sequestosome 1; LC 3: microtubule-associated protein light chain 3).

Mitochondrial quality control is an elaborate and complicated network in eukaryocytes for maintenance of mitochondria homeostasis in eukaryotes by means of four core processes: mitochondrial proteostasis, biogenesis, dynamics and autophagy. Mitochondrial dysfunction amplified by defective quality control processes is gradually regarded as the major pathophysiologic mechanism of sarcopenia (Fig. 1).

### 2.1 Mitochondrial Proteostasis

Mitochondrial proteostasis plays an essential role in retaining the dynamic balance between new protein synthesis and impaired protein degradation. Imbalanced proteostasis leads to the accumulation of unnecessary and defective proteins, which further adversely impacts multiple physiological functions, including skeletal muscle activity [36].

An intricate mechanism for the removal of misfolded and dysfunctional proteins is present in muscle mitochondria. The ubiquitin-proteasome system (UPS) and autophagy-lysosome system (ALS) are two predominant pathways to selectively eliminate damaged mitochondrial proteins [37, 38]; UPS mainly eliminate single and unfolded proteins under the tight regulation of AMP-activated protein kinase (AMPK) and the Forkhead Box O (FoxO) transcription factor family [37, 39]. FoxO3 can decrease skeletal muscle mass directly by activating the downstream muscle-speciﬁc ubiquitin ligases, the Muscle RING Finger 1 (MuRF1) and the Muscle Atrophy F-box (MAFbx), that are considered as the key regulators of protein turnover contributing to muscle atrophy [40, 41]; FoxO3 can also act indirectly through inactivating the mammalian target of rapamycin complex 1 (mTORC1), a pro-anabolic regulator of protein production [42]. On the other hand, diverse mitochondrial stress conditions associated with the aging process stimulate the mitochondrial unfolded protein response (UPRmt) that serves as a central node to restore proteomic homeostasis [43, 44]. When nascent polypeptides are perturbed during the import and fold processes, mitochondria-specific chaperones, mitochondrial proteases (mitoproteases) and the UPRmt are initiated to repair these “errors”. Chaperones assist with protein import into mitochondria and are responsible for folding of unfolded or misfolded proteins [45, 46], while mitoproteases form the first line of defense against cellular stress responses by degrading irreversibly damaged proteins [47]. Intriguingly, numerous stress responses simultaneously activate the UPRmt and mitophagy [48]. In this scenario, UPRmt serves as the basal folding and degradation mechanism to counteract mitochondrial protein dyshomeostasis, and mitophagy can completely eliminate the unsalvageable mitochondria suggesting that UPRmt-dominated protein degradation and mitophagy are complementary processes [48].

The synthesis of new and properly functioning proteins is an important part of protein turnover in mitochondria. An increasing number of extensively studied regulators of protein synthesis in skeletal muscle are likely to be associated with the pathogenesis of sarcopenia. Akt is the key element of the PI3K/Akt/mTOR signaling pathway and acts as a pivotal mediator in the homeostasis of muscle mass through the mTOR/p70S6K and FoxO3/MuRF-1 and MAFbx pathways to regulate protein synthesis and degradation, respectively [49]. Moreover, AMPK can abrogate the protein synthesis in skeletal muscle by suppressing the mTOR signaling pathway and activating the FoxO-dependent degradation pathway [50]. Furthermore, peroxisome proliferative activated receptor-γ coactivator-1α (PGC-1α) and its variants can stimulate protein synthesis and mitigate muscle protein degradation by UPS through repressing the activities of nuclear factor κB (NF-κB) and FoxO3 activity [51-53]. PGC-1α knockout mice manifested a failure of skeletal muscle function partially due to UPRmt dysregulation [54].

Protein dyshomeostasis may induce muscle mass and strength insufficiency in sarcopenia. UPRmt-related genes were significantly decreased in the muscle of sarcopenia patients, including those encoding mitochondrial heat shock proteins (HSP) and proteases [25]. In addition, mTOR signaling pathway, an important hallmark of mitochondrial protein anabolism, was restrained in sarcopenic participants as well [25]. In the skeletal muscle of senescence-accelerated mouse (SAM) prone 8 (SAMP8), a canonical sarcopenia model, protein synthesis-related markers (Akt, mTOR and p70S6K) were reduced; however, the protein degradation-associated markers (FoxO3, MuRF-1 and MAFbx) were elevated indicating during advancing process of aging, protein turnover has a pro-degradation trend that leads to muscle atrophy which contributes to the occurrence of sarcopenia [55]. A study indicated that the activation of FoxO and proteolytic systems was not involved in sarcopenia, but mTORC1 overactivity was found in aged mice [56, 57]. However, these results were not remarkably observed in human individuals. In addition, rapamycin, an inhibitor of mTORC1, ameliorated sarcopenic symptoms, including less age-related loss of muscle mass and improved muscle functions [57]. Therefore, the functional roles of FoxO and mTORC1 in sarcopenia are controversial and need further investigations. Imbalanced mitochondrial proteostasis significantly reduces the muscle mass and compromises mitochondrial biogenesis resulting in the dysfunction of mitochondria and skeletal muscle. However, the extent of associations of imbalanced mitochondrial proteostasis in senescent muscle with sarcopenia requires additional investigations.

### 2.2 Mitochondrial Biogenesis

Mitochondrial biogenesis is a multistage process that generates new mitochondria [19]. Mitochondrial proteins are encoded by the nuclear and mitochondrial genomes. The nuclear genome encodes most of proteins involved in mitochondria genesis, whereas mitochondrial DNA (mtDNA) encodes a small number of crucial subunits of the electron transport chain (ETC) complexes. Mitochondrial biogenesis mainly comprise three steps: transcription of nuclear genes, import of nuclear-encoded mitochondrial proteins (NEMPs), and transcription and replication of mtDNAs [58]. PGC-1α is the main factor in regulating mitochondrial biogenesis in the cooperation with downstream nuclear transcription cofactors [38, 59], such as nuclear respiratory factor-1 and -2 (Nrf-1 and Nrf-2) and estrogen-related receptor alpha (ERRα) [59]. Once activated, Nrf-1 and Nrf-2 cofactors bind to the target nuclear genes and promote the expression of NEMPs and mitochondrial transcription factor A (Tfam), which can directly bind to target mtDNA and activate the replication and transcription of the corresponding regions of mtDNA [58, 60].

Mitochondrial biogenesis and its regulators collectively participate in the pathophysiologic changes in sarcopenia. In an interethnic study of human sarcopenia, it was demonstrated that expression profiles of PGC-1α, ERRα and other coactivators were reduced in sarcopenic individuals [25]. PGC-1α, Nrf-1 and Tfam are downregulated in SAMP8 mice during the onset and development of sarcopenia [55]. Moreover, a pronounced decline in muscle mass, muscle performance and frailty was observed in old Nrf-2 knockout (Nrf-2 KO) mice with decreased expression levels of PGC-1α, Nrf-1 and Tfam, suggesting that knockout of Nrf-2 may exacerbate skeletal muscle frailty and sarcopenia through compromising mitochondrial biogenesis [61]. In addition to prototypical cofactors of mitochondrial biogenesis, Zhou and colleagues [62] demonstrated that deficiency of high-temperature requirement protein A2 (HtrA2/Omi) protease is involved in the development of sarcopenia by negatively regulating mitochondrial biogenesis. HtrA2/Omi is a quality control protease that mainly localizes in the intermembrane space of the mitochondria [63]. HtrA2 ^mnd2 (-/-)^ mice manifested low muscle mass, poor physical function and sarcopenia phenotypes. Intriguingly, HtrA2 apparently regulates mitochondrial biogenesis in sarcopenia via the differential expression of Nrf-1/2, but not PGC-1α.

PGC-1α is a critical regulator that maintains muscle homeostasis and has attracted considerable attention due to the studies of its potential effects in aging-associated diseases [17]. In mammalian cells, PGC-1α serves as a nuclear-mitochondrial hub in mitochondrial biogenesis [64] by translocating from the cytosol to the nucleus and mitochondria [64, 65]. Muscle atrophy and poor exercise performance combined with reduced PGC-1α levels in skeletal muscle have been detected in the elderly population [66], and the mRNA and protein expression levels of PGC-1α were considerably decreased in the soleus muscle of old rats, indicating that PGC-1α downregulation may participate in the course of aging [67]. The expression levels of PGC-1α, Tfam and Nrf-1 were decreased in the muscle of old SAMP8 mice [55] and elderly individuals [66, 68]. In contrast, PGC-1α overexpression can counteract the loss of muscle mass [69] and muscle atrophy [52] through regulating the homeostatic mechanisms of MQC, although it has not been found in the process of muscle aging. Notably, three prominent metabolic regulators (AMPK, PGC-1α and SIRT1) apparently cooperate to hinder the progression of sarcopenia. AMPK plays a crucial regulatory role in mitochondrial biogenesis [70, 71] and its biological activity in mitochondrial biogenesis declines with aging [72]. Activation of the AMPK/PGC-1α pathway facilitates mitochondrial biogenesis in skeletal muscle [73-75]. Moreover, silent mating type information regulation 2 homolog sirtuin 1 (SIRT1), an NAD^+^-dependent deacetylase [63], can directly deacetylate and activate PGC-1α in the cytoplasm. SIRT1 colocalizes with PGC-1α in the mitochondria and serves as a downstream regulator of AMPK in response to exercise and fasting [76]. It has been shown that the AMPK/SIRT1/PCG-1α pathway protects the heart from aging and stress [77]. In sarcopenia patients, the NAD^+^ levels was reduced [25], while AMPK can elevate NAD^+^ levels and thus activate NAD^+^-dependent SIRT1 [25, 78]. Moreover, activated SIRT1 deacetylates PCG-1α and induces its expression to enhance mitochondrial synthesis, assembly, growth and antioxidant capability in the heart [77]. It is thus reasonable to suggest that AMPK, PGC-1α and SIRT1 form an integrated and coordinated pathway to attenuate impaired mitochondrial biogenesis associated with aging. Considering the important role of PGC-1α in MQC, the pathway may become a promising target for the prevention and treatment of sarcopenia.

### 2.3 Mitochondrial Dynamics

Mitochondria are highly dynamic organelles that constantly fuse with surrounding mitochondria and split into daughter mitochondria [79]. Mitochondrial dynamics involves two processes (fusion and fission) [20] that are indispensable for mitochondrial maintenance [79]. Fusion and ﬁssion enable the efﬁcient distribution and exchange of mitochondrial contents to meet the local demands of the cells [80]. Mitochondrial fusion is a complementary process that mixes the contents of damaged mitochondria under multiple stress conditions, and mitochondrial fission is a divisive process that results in the production of new mitochondria and contributes to the homeostasis of MQC by removing malfunctioning mitochondria and promoting apoptosis [81]. A family of conserved large GTPases are essential for the regulation of mitochondrial dynamics, such as mitofusins 1 and 2 (Mfn1, Mfn2) and optic atrophy 1 (Opa1) for mitochondrial fusion, and dynamin-related protein 1 (Drp1), mitochondrial ﬁssion factor (Mff) and ﬁssion protein 1 (Fis1) for mitochondrial fission [82-84]. Mitochondrial dynamics can repair mild damage to mitochondria and is associated with autophagy to thoroughly eliminate impaired mitochondria, especially through mitochondrial fission [81]. Imbalanced mitochondrial dynamics is a common hallmark of senescence [85] that can lead to mitochondrial swelling, decreased cristae production and defective respiratory function [86] thus severely affecting cellular homeostasis.

Several proteins that mediate mitochondrial dynamics are found to be dysregulated in skeletal muscle. For instance, Drp1 knockdown was found to induce muscle atrophy [87, 88]. Currently, the great majority of researches demonstrated that mediators relevant to mitochondrial dynamics are decreased or deficient in aged muscle. Paucity of Mfn2 [89] and deletion of Opa1 and Drp1 [90] have been shown to lead to skeletal muscle atrophy in mice of advanced age. Mitochondrial fusion- and fission-related proteins are differentially expressed in mice and humans with sarcopenia or sarcopenic symptoms. Decreased mRNA expression levels of Mfn1, Mfn2 and Opa1 were detected in the skeletal muscle of elderly mice with sarcopenic phenotypes [91]. In another study, the expression levels of Mfn2, Fis1 and Opa1 were downregulated in sarcopenic muscle, suggesting that mitochondrial dynamics participates in the pathogenesis of sarcopenia [25]. Similarly, mitochondrial fusion genes (Mfn2 and Opa1) were substantially downregulated in old SAMP8 mice and demonstrated a downward trend during the whole progression of sarcopenia [55]. In sarcopenia patients, Opa1 expression was reduced during senescence [92]. Mfn2 protein expression was also markedly decreased in hip-fractured patients with sarcopenia [93]. It is universally appreciated that mitochondrial fission plays an indispensable role for the elimination of impaired mitochondria by mitophagy, whereas some studies have shown that the mitochondrial dynamics is shifted toward fusion in sarcopenia. Intriguingly, fusion-related proteins (Mfn1/Mfn2) were significantly upregulated in older wild type (WT) mice due to the downregulation of fission protein Fis1 [94], which is consistent with the results obtained in some hip-fractured patients of advanced age with sarcopenia [95]. In addition, Huang et al. [61] identified that mitochondrial fusion-related factors (Mfn1, Mfn2 and Opa1) and mitochondrial fission-related factor (Drp1) were decreased in old Nrf-2 KO mice with sarcopenia, suggesting that loss of Nrf-2 can aggravate sarcopenia by disrupting balanced mitochondrial dynamics and that mitochondrial biogenesis and dynamics may be associated with each other.

These aforementioned studies demonstrated the prominent role of abnormal mitochondrial dynamics in muscle aging and sarcopenia (Table 1); however, the mechanisms of these processes remain poorly understood. The alternations in mitochondrial fusion and fission per se are closely associated with mitophagy and mitobiogenesis to form a finely tuned network that prevents and repairs mitochondrial damage. Therefore, investigation of the molecular mechanism of this process is needed, and effective interventions to delay and even reverse the progression of sarcopenia should be developed.

**Table 1:** Mitochondrial dynamics-related factors in sarcopenia.

	Key Regulators	Biofunction	Expression in Sarcopenia	Reference
**Mitochondrial Fusion**	Mfn1, Mfn, and Opa1	Mixing the contents of impaired mitochondria	Downregulated	[55, 61, 92, 93]
**Mitochondrial Fission**	Drp1, Mff, and Fis1	Generating daughter mitochondria, removing damaged mitochondria and promoting apoptosis	Downregulated	[61, 94, 95]

### 2.4 Mitochondrial Autophagy

Entire mitochondrion can be degraded by autophagosomes with a fused lysosome through an intricate catabolic process termed autophagy. Mitophagy is an exceptional type of macroautophagy that primarily mediates the selective removal of the damaged or superfluous organelles and protein aggregation to maintain mitochondrial homeostasis [96, 97]. Traditionally, the activation of the ULK1-Atg13-FIP200 complex is considered as the canonical initiator of autophagy that culminates in the generation of a double-membrane autophagosome, engulfment of cellular proteins and organelles and fusion with lysosomes [98, 99]. Recently, the PINK1/Parkin pathway has been recognized as one of the most important signaling pathways that regulates ubiquitin-dependent mitophagy [100]. When the membrane potential of damaged mitochondrion is depolarized, the import of PINK1 into IMM is inhibited and the protein accumulates on the outer mitochondrial membrane (OMM) inducing the recruitment of Parkin from the cytosol to the OMM [96, 101-103]. Parkin is an E3 ubiquitin ligase, and its activity is triggered by the PINK-dependent phosphorylation [104, 105]. Then, activated Parkin ubiquitinates outer membrane proteins, generating ubiquitin (Ub) and poly-ubiquitin (poly-Ub) chains. Poly-Ub chains are subsequently phosphorylated by PINK1 and serve as an autophagic signal. Ubiquitin-binding adaptor proteins, including p62, optineurin (OPTN) and nuclear dot protein 52 (NDP52), recognize phosphorylated poly-Ub chains on mitochondrial proteins and recruit damaged mitochondria to the isolation membrane through their interaction with microtubule-associated protein light chain 3 (LC3) [96, 103]. Finally, the damaged mitochondrion is engulfed by an autophagosome that can further fuse with a lysosome to form an autolysosome thus eliminating the entire mitochondrion. In addition to the PINK1/Parkin pathway, important mitophagy receptors (NIX [106], BNIP3 [107, 108] and FUNDC1 [109]) can localize to OMM and directly bind to LC3 to recruit autophagosomes and facilitate mitochondrial elimination as well [103].

Biological aging adversely affects mitophagy homeostasis in various organs and systems [110-112] and impaired mitophagy plays a crucial role in the loss of normal mitochondrial functions [111]. Certain alternations in numerous mitophagy regulators have been reported in senescent animals and humans [94, 113] to lead to mitophagy deficiency and subsequent accumulation of malfunctional mitochondria in skeletal muscle with aging [114, 115]. The accumulation of damaged mitochondria causes the dysfunction of skeletal muscle cells accompanied by muscle wasting and muscle strength reduction [116, 117]. Therefore, insufficient mitophagy potentially plays a causative role in sarcopenia. During the course of sarcopenia progression in SAMP8 mice, elevated Atg13 and LC3-II levels were associated with the accumulation of p62 and lysosome-associated membrane protein 1 (LAMP1), suggesting that poor fusion between autophagosomes and lysosomes and impaired mitophagy in sarcopenic mice [55]. In another premature aging model with sarcopenia, old WT mice had lower expression levels of beclin-1 and p62 than those in young mice, indicating an autophagic dysfunction in aging muscle [94]. The downregulated expression of the autophagy mediator, LC3B, has also been detected in muscle from elderly hip-fractured patients with sarcopenia as well [93]. Recently, many studies have demonstrated that Parkin deletion results in inadequate muscle mass and poor physical performance in old individuals and mice [114, 118], whereas Parkin overexpression has a protective effect on skeletal muscle [118]. Similarly, Leduc-Gaudet el al. [119] have demonstrated that Parkin overexpression can ameliorate the reduction in muscle mass and strength during senescence. The authors demonstrated that after intramuscular injection of adeno-associated virus (AAV) vectors for encoding Parkin induced the upregulation of Parkin expression and attenuated the loss of skeletal muscle mass and strength in old mice versus those in the control group. Moreover, mitochondrial derived vesicles (MDVs) function as important mediators in the vesicle transport between mitochondria and lysosomes, which was regarded as an Drp1-independent mitophagy pathway [120]. And MDV-derived nicotinamide adenine dinucleotide may become a novel biomarker for sarcopenia [121].

Additionally, mitochondrial autophagy, dynamics and biogenesis are closely associated with each other. Mitophagy and mitochondrial dynamics are interrelated because the conversion between fusion and fission is a prerequisite for mitophagy [79]. PINK1 can indirectly activate Drp1 to promote the degradation of defective mitochondria [122]. Parkin induces the proteasomal degradation of mitofusins thus shifting the mitochondrial fusion/ﬁssion balance towards ﬁssion to suppress mitochondrial fusion and induce segregation of malfunctioning mitochondria from the healthy mitochondrial network [123]. Besides, Parkin is positively associated with mitochondrial biogenesis due to proteasomal degradation of Parkin-interacting substrate (PARIS), a zinc-finger protein that inhibits the synthesis and secretion of PGC1α and blunts the activation of its target cofactors and genes [124]. These findings indicate that mitophagy may contribute to sarcopenia through a complex MQC network. Considering multiple roles of mitophagy in quality control, it is deserved to expect that Parkin or other important regulators of mitophagy may become novel targets for the prevention and attenuation of sarcopenia during aging.

In summary, the maintenance of homeostatic MQC is attributed to the synergistic regulation of mitochondrial proteostasis, biogenesis, dynamics and autophagy in senescence. Disruption of quality control homeostasis results in accunulation of dysfunctional mitochondria that negatively affects skeletal muscle health in older individuals and may even lead to sarcopenia.

## 3. Potential Interventions for Sarcopenia

### 3.1 Exercise Therapy

Physical activity, especially resistance exercise training, has been recommended as the first-line intervention to manage sarcopenia according to the evidence-based clinical practice guidelines [35].

#### 3.1.1 Resistance exercise for sarcopenia

Physical activities that set off skeletal muscle contraction against resistance can be defined as resistance exercise. Resistance exercise increases muscle mass, elevates muscle strength and improves the performance of physical exercise in elderly sarcopenia patients [35, 125, 126]. Several studies have shown that resistance exercise promotes mitochondrial protein synthesis and thus plays a role in amelioration of mitochondrial dysfunction [127, 128] to improve muscle mitochondrial biofunctions in human skeletal muscle [129]. Resistance exercise has been shown to optimize mitochondrial functions, particularly by improving mitochondrial biogenesis. Specific molecular mechanisms mainly include the AMPK/SIRT1/PGC-1α or FoxO1 axis as regulators of mitochondrial biogenesis in response to physical exercise of skeletal muscle [76]. In such a scenario, exercise training activates AMPK due to an increase in the AMP/ATP ratio [130] which directly phosphorylates PGC-1α. Subsequently, activated PGC-1 translocates from cytosol into the nucleus and coactivates the transcription factors and nuclear receptors to enhance mitochondrial biogenesis [65, 131]. Besides, long-term moderate exercise positively regulates mitochondrial biogenesis through coordinated interactions of AMPK, SIRT1 and PGC-1α in the skeletal muscle of older mice [132]. In addition to the effects on mitochondrial biogenesis, several studies have demonstrated that exercise training also contributes to mitochondrial homeostasis by preserving mitophagy in skeletal muscle, which may potentially benefit patients with sarcopenia [133, 134]. Interestingly, voluntary resistance wheel exercise (RWE) has been warranted as an effective way to prevent sarcopenia in old C57BL/6J mice [135]. Exercised sarcopenic mice manifested improved mitophagy (increased LC3II/I ratios) and mitochondrial functions (higher mitochondrial density and better oxidative capacity) compared to those in 23-month-old sedentary controls, and the differences were not sex-specific.

#### 3.1.2 Endurance training for sarcopenia

In addition to resistance-based exercise, endurance training is involved in the maintenance of MQC in skeletal muscle as well, including mitochondrial protein synthesis [127, 136, 137], mitophagy [138, 139] and also general functions [137], which likely contribute to the prevention and management of sarcopenia during aging. Endurance exercise promoted the clearance of impaired proteins in skeletal muscle in addition to the prosynthetic effects of physical activity [140]. But the clinical significance of endurance exercise on managing sarcopenia requires further investigation.

Current studies indicate that physical exercises enhance muscle performance and relieve sarcopenic manifestations, in part due to optimized MQC. Currently, consistent recommendation of specific physical exercise for sarcopenia patients is not available; however, exercise plans should correspond to patient abilities and rehabilitation goals to achieve the best therapeutic effect.

### 3.2 Nutritional and Pharmacological Interventions

Certain limitations for physical exercises in patients with sarcopenia include increased risk of fractures and falls in older adults and poor adherence to long-term training programs [141]. Therefore, valid nutritional and pharmacological interventions have to be identified to ameliorate sarcopenia in a more easily available fashion. Currently, clinically approved drugs for specific treatment of sarcopenia are unavailable; however, a few medicines are undergoing phase I and II clinical trials [4]. The supplementary nutrients mainly include protein, vitamin D, antioxidants, myostatin inhibitors and anabolic hormones [4, 35, 142]. Nutritional manipulations demonstrated certain efficacy in the improvement of the symptoms of sarcopenia; however, none of these manipulations are recommended as conventional methods for therapy of sarcopenia, except protein intake, which is conditionally recommended [35].

Recently, several bioactive compounds or drugs have been identified as latent and potent options to prevent and delay the progress of sarcopenia through ameliorating mitochondrial dysfunction. Melatonin (N-acetyl-5-methoxytryptamin, aMT) is a pineal hormone that is ubiquitously present in most organs and tissues, including skeletal muscle [143]. Melatonin coordinates physiological adaptations to the light/dark cycle and seasonal alterations and has a number of other biofunctions, such as recovery of aging-related mitochondrial dysfunction of muscle in senescence-accelerated mice [143-145]. Notably, a protective effect of melatonin in sarcopenia is mediated by the modulation of mitochondrial changes in aging. Sayed et al. [146, 147] demonstrated that oral melatonin treatment of early-aged (12 months) mice with sarcopenia resulted in relatively normal muscle structures, increased number of muscle fibers, decreased frailty index (FI) and improved physical performance. Additionally, melatonin supplementation promoted lactate production and diminished tubular aggregate formation and nuclear apoptosis. These results indicated that melatonin plays the prophylactic role in sarcopenia during aging, which should probably be used in clinical therapy in the near future. In addition to endogenous hormones, 5,7-dimethoxy?avone (DMF), a major ?avone detected in Kaempferia parvi?ora, was demonstrated to serve as a natural ingredient to delay sarcopenia [148]. In this study, oral administration of DMF considerably increased muscle mass, strength, and physical ability and three basal evaluation indexes of sarcopenia compared to those in the aged controls. At the molecular level, DMF regulated protein synthesis by stimulating the PI3K-Akt axis and mTOR pathway and restraining the FoxO pathways and enhanced mitochondrial biogenesis by upregulating the mRNA expression of PGC-1α, Nrf-1, and Tfam. Another natural substance, oligonol, has been demonstrated to increase muscle mass and strength by optimizing the quality control of mitochondria in SAMP8 mice [149]. Oligonol is extracted from ?avanol-rich lychee and can regulate metabolism [150, 151]. After 8 weeks of oligonol administration, SAMP8 mice in the experimental group had higher skeletal muscle mass and strength versus those in the control animals. At the molecular level, oligonol positively regulated mitochondrial proteostasis (stimulation of Akt/mTOR/p70S6K and inhibition of FoxO3a/MuRF1 and MAFbx signaling), mitochondrial biogenesis (elevated PGC-1α and Tfam), mitochondrial dynamics (increased Mfn2 and Opa1) and mitophagy (upregulation of PINK1, a reduction in Atg13, LC3-II, and p62, and a decrease in autophagosomes and lysosomes), indicating that oligonol can play a role in the correction of mitochondrial dysfunctions thus preventing sarcopenia. Notwithstanding, the underlying mechanisms derived from aging-accelerated mice may differ from normally senescent mice. Additionally, 5-aminolevulinic acid (ALA), a basic compound in porphyrin synthesis, was demonstrated to reduce the loss of muscle mass and improve physical performance in old mice through facilitating protein synthesis in mitochondria [152]. The decreased level of NAD^+^ was detected in sarcopenia, cardiovascular aging and neurodegenerative diseases [25, 78, 153]. Nicotinamide, an NAD+ precursor, has been shown to promote mitophagy and suppress cardiac aging through activating Sirtuins [154, 155]. Therefore, targeting NAD^+^ may become a novel strategy for alleviating sarcopenia. Moreover, urolithin A, as one of the end-products of ellagitannins (ETs) and ellagic acid (EA), was also found to induce mitophagy and improve muscle functions in aged mouse, which probably becomes a promising nutritional supplementation for sarcopenia [156]. However, all of these bioactive compounds or drugs are carried out in animal models, and none of them are tested in clinical trials.

Overall, the combination of physical exercise and protein supplementation is the most effective countermeasure for sarcopenia. However, nutritional and pharmacological interventions are more applicable for the majority of sarcopenia patients. Several potential therapeutic agents have been demonstrated to mitigate mitochondrial malfunction for the prevention and treatment of sarcopenia. Furthermore, gene therapy probably become a novel strategy for alleviating sarcopenia. METTL21c acts as a bone-muscle pleiotropic gene for sarcopenia. Although METTL21c’s biofunction is achieved by NF-κB signaling pathway, its potential role in quality control mechanisms of mitochondria deserves more investigation [157]. Additionally, some mitokines potentially function as biomarkers for the diagnosis of sarcopenia, such as growth differentiation factor 15 (GDF15) and fibroblast growth factor 21 (FGF21) [158, 159]. Therefore, an increasing number of studies are needed to confirm the effectiveness of the present findings and to explore novel countermeasures and diagnostic methods for sarcopenia, which may greatly improve the quality of life of elderly patients.

## 4. Conclusion

Sarcopenia is a prevalent and degenerative skeletal muscle disease that leads to poor quality of life in patients, particularly elderly individuals. Numerous pathophysiological alterations contribute to a progressive decline in muscle strength, muscle mass and physical performance, which are the predominant hallmarks of sarcopenia. Dyshomeostasis of mitochondrial quality control is one of the primary factors for the initiation and progression of sarcopenia. MQC requires coordination of mitochondrial proteostasis, biogenesis, dynamics and autophagy. Alterations in mitochondrial quality control exacerbate muscle atrophy and reduce muscle strength during aging concomitant with restricted locomotive function. The important regulators and signaling pathways in MQC may be involved in the etiology of sarcopenia and are comprehensively summarized in this review. Physical exercise, the most recommended therapeutic intervention for sarcopenia, improves skeletal muscle quantity and quality partially by facilitating the restoration of malfunctional mitochondria. Additionally, nutritional and pharmaceutical treatments ameliorate sarcopenia to a certain degree, although compelling evidence and mechanisms remain to be identified. Overall, dysfunctional MQC plays a causative role in sarcopenia and revealing latent mechanisms may shed light on efficient preventive and intervention strategies for patients with sarcopenia.
